# Evaluation of an online training course for educational professionals on depression and mental health in childhood and adolescence

**DOI:** 10.1186/s13034-025-01007-y

**Published:** 2025-12-15

**Authors:** Ann-Kathrin Saffenreuther, Ellen Greimel, Regine Primbs, Lucia Iglhaut, Sara Kaubisch, Gerd Schulte-Körne, Lisa Feldmann

**Affiliations:** 1 Department of Child and Adolescent Psychiatry, Psychosomatics and Psychotherapy, LMU University Hospital, Ludwig-Maximilians-University (LMU) Munich, Nussbaumstr. 5, 80336 Munich, Germany; 2 German Center for Mental Health (DZPG), Partner Site Munich-Augsburg, Munich, Germany

**Keywords:** Depression, Training, Evaluation, School intervention, Prevention, Teachers

## Abstract

**Background:**

The rising rates in depression and other mental health problems among adolescents in recent years, combined with delayed help-seeking, highlights a clear need for action in key areas of adolescents’ lives. The school environment, including teachers, can provide mental-health support for children and adolescents.

**Methods:**

The study used a pre-post-follow-up design to investigate whether a two-hour online training course on depression and mental health in childhood and adolescence leads to knowledge acquisition, confidence in supporting pupils with depression and a decrease in stigmatisation in *N* = 97 teachers, school social workers and school social pedagogues.

**Results:**

We found a significant increase in knowledge and confidence in supporting pupils with depression at post-assessment, which was maintained at a four-week follow-up. A decrease in stigmatisation was found at post-assessment, which was no longer evident at follow-up.

**Conclusion:**

The results suggest that approaching educational professionals through a concise practical online training course can be a promising strategy for knowledge transfer. Future studies could examine whether such trainings can be adapted to other mental health disorders.

*Trial registration* ClinicalTrials.gov, Identifier: NCT06387108. Registered on 24.04.24.

**Supplementary Information:**

The online version contains supplementary material available at 10.1186/s13034-025-01007-y.

## Background

Major depression is a severe mental disorder that often begins in childhood and adolescence with a lifetime prevalence of about 19% in adolescence [1, 2]. Despite being among the most common mental disorders in adolescents [3], depressive disorders yet often remain unrecognised and untreated for a long time [4]. The associated costs with major depression are high, including limitation in social functioning [5], an increased risk of developing comorbidities [6], high direct healthcare costs (i.e. inpatient and outpatient treatment, emergency care, medication; [7]), and reduced quality of life [8]. Adolescence and young adulthood are characterized by significant developmental changes and increased vulnerability to psychological disorders [2]. According to the diathesis-stress model, the development of depression emerges as a result of the interaction between predisposed factors (diatheses) and stressors [9]. School stressors, such as high-performance expectations from parents and teachers or bullying experiences, have been identified as crucial factors in the development and maintenance of depression in pupils [10–12]. At the same time, mental illness is one of the main reasons for dropping out of school [13]. A large number of pupils with mental health problems, including depression do not seek mental health care [14]. Professional help is usually only sought once the mental illness has reached a high level of severity [15]. School, as a central area of life for children and adolescents, is an important place where pupils’ mental health can be promoted by enhancing a pattern of positive coping [16].

### Knowledge, confidence and stigma

One barrier to seek professional help is the anticipated stigmatisation of those in need for help [15]. People with depression compared to non-depressed people are prototypically described by laypeople as more overweight, less self-caring, less successful, less attractive and more introverted [17]. Youth perceive high barriers for seeking help and, additionally, the lack of treatment options can lead to longer delays or even prevent necessary care [14]. Schools have the potential to support pupils’ mental health by reaching all pupils – including those who might otherwise not access the mental health care system [18]. The limited implementation of early mental health support by school staff can be attributed to a lack of knowledge among teachers and a lack of confidence in their ability to support pupils with mental health problems [19, 20]. In the school context, it is particularly difficult to recognise pupils with internalising disorders such as depression [21]. Mental health literacy and confidence in one’s own competence in helping someone are considered predictors of the helping behaviour shown towards pupils [22]. The increase in depressive disorders in adolescence observed in recent years and the consistently late timing of interventions (such as consulting and psychotherapy) show a clear need for action in the everyday lives of adolescents [18]. Providing access to evidence-based information on depression and mental health through training for educational professionals in schools is intended to help to reduce the large treatment gap.

### Current state of research on training courses for educational professionals on depression and mental health

Systematic reviews on mental health training programmes for school teachers showed an improvement in teachers’ knowledge about mental health/ illness and reduced stigma towards mental illness after the intervention and at the follow-up [23, 24]. There was also evidence of an increase in (helping) behaviour and confidence in one’s own abilities in supporting adolescents with depression [24].

Most existing teacher training courses take place in person and over several days (for example see [25, 26]). The number of evaluated teacher trainings in an online format is small, though an online format provides the opportunity to increase the willingness to participate and to reduce time and financial costs [27]. A positive example that targeted mental health in general and not depression specifically can be found in the studies by Green [28] and Albright [29] conducted in the U.S., which demonstrated enhancements in teachers’ preparedness, confidence, and gatekeeper behaviours in recognising and supporting pupils with mental health issues. Short training sessions, combined with a targeted focus on strategies for teachers to support adolescents within the school setting, are essential to reach a broad range of educational professionals [30]. Teacher training programmes mostly focus on mental health in general, and only a few target depressive disorders specifically [19, 25, 31]. Research in German-speaking countries, particularly Germany, where the present study was conducted, indicates that teachers exhibit significant gaps in action-oriented knowledge for supporting pupils with mental health disorders [32]. Moreover, there is a clear lack of evidence-based, evaluated trainings for educational professionals on mental health/depression, which could help to address this gap [31]. Coppens [19] recorded the effects of a training on depression knowledge, stigma towards depression and confidence in one’s own ability to recognise suicidal tendencies in social professions (teachers, nurses and social workers) in Germany, Ireland and Portugal. Teachers demonstrated low initial knowledge about suicide and limited confidence in identifying suicidal individuals before the training and showed large training effects. Overall, the study showed stable improvements in knowledge, confidence and reduction in stigma over time across professions and countries [19]. Grabowski [31] evaluated an online teacher training programme on depression in a few teachers and mostly pre-service teachers and found significant increases in knowledge acquisition, action competence, and self-perceived competence in supporting pupils with depression. The changes remained stable over a 1-month follow-up [31].

By imparting knowledge, reducing stigma and building confidence, educational professionals should be sensitised to depressive illness so that they can better respond to pupils with depression and initiate appropriate support services. Therefore, the aim of the current study was to evaluate a short online training course on depression and mental health for educational professionals in schools.

Based on previous studies [23, 24, 33], it was hypothesised that participation in the training would lead to (a) an increase in knowledge regarding depression in youth, (b) a decrease in stigma towards depression and (c) an increase in confidence in one’s own ability to support pupils with depression after the training and at a four-week follow-up. Moreover, it was hypothesised that (d) reported helping behaviour would increase at the four-week follow-up.

## Methods

A pre-post-follow-up design in a sample of educational professionals from Lower Saxony (Germany) was employed to investigate the effects of a two-hour online training course for educational professionals on depression and mental health in childhood and adolescence. This pilot project was conducted by the Department of Child and Adolescent Psychiatry, Psychosomatics and Psychotherapy of the LMU University Hospital Munich, together with the Beisheim Foundation. The study protocol was pre-registered through ClinicalTrials.gov (identifier: NCT06387108) and has been performed in accordance with the Declaration of Helsinki and approved by the local ethics committee.

### Participants

A total number of *N* = 97 (*n* = 87 female, *n* = 10 male) educational professionals, aged 27 to 63 (*M* = 41.4, *SD* = 9.3) were included in the study. *N* = 71 worked as teachers and *n* = 26 as school social workers and school social pedagogues (in the German school system, they provide school-based counselling and support for personal and social challenges). A total of 124 individuals registered for the study, 97 actually participated in the pre-test, 95 in the post-test and 79 in the follow-up. Individuals who participated in none or only one assessment point were excluded from the analyses, resulting in an overall dropout rate of 21.77% (*n* = 27; these participants are not part of the *N* = 97 total number of participants described above). The inclusion criterion was a current activity in the teaching profession (teacher) or a profession in school. German language skills were presumed, as the training and evaluation took place in German. Information on participants’ demographics was received via self-report in a self-designed sociodemographic questionnaire. Of the 71 teachers, 18 were working in primary school (“Grundschule”), 5 in secondary general school (“Mittelschule”), 3 in intermediate school (“Realschule”), 19 in grammar school (“Gymnasium”), 17 in comprehensive school (“Gesamtschule”), 6 in vocational school (“Berufsschule”), and 3 in special-needs school (“Förderschule”), with a mean of 11.03 years of work experience (*SD* = 7.79). As regards their experience with pupils with psychological problems, 41 educational professionals reported being currently in contact with one or more pupils with diagnosed depression, 27 assumed they had been engaging with a pupil with depression, and 17 suspected they had been interacting with pupils with other types of mental disorders. Participants were informed about the training course and study by the Lower Saxony Ministry of Education via an official press release. Additionally, educational professionals in Lower Saxony were sent an e-mail containing the possible participation time slots and the login details for the online training course, along with a separate link to register for the current study. Through a collaboration with the Lower Saxony Ministry of Education, the project of the ministry included both, a six-week MHFA course and our online training course. Therefore, prior to participating in our training and study, all participants took part in a six-week Mental Health First Aid (MHFA) training programme. The MHFA course consists of six online courses each lasting two hours on mental health and mental disorders (anxiety disorders, psychosis, depression, substance use disorders) in adulthood. MHFA is a separate training programme that teaches participants to recognise mental health issues among adults, provide immediate support, and direct individuals to professional help [34]. The MHFA programme has been evaluated in other studies [35]. The average duration between the MHFA course and our training was 23 weeks (*M* = 22.76, *SD* = 12.81). The combination of the MHFA and our training course was planned together with the Lower Saxony Ministry of Education to first provide foundational knowledge on adult psychiatric disorders, followed by school-focused practical knowledge on supporting children and adolescents with depression. To avoid content overlap, all materials were pre-coordinated with the MHFA course trainers. Regarding depression-related content, the MHFA course addressed, for example, how affected colleagues can be supported. In contrast, our online training course on depression and mental health in children and adolescents focused on age-dependent symptom presentation with differentiation from pubertal signs, communication within the classroom, interaction with parents of pupils with depression, and information about compensatory measures in the school context to support pupils in meeting academic requirements without being disadvantaged. To evaluated solely our training session and not the effects of MHFA, the pre-test time point was always after the last MHFA session.

### The training

The two-hour online training for educational professionals in schools was intended to provide low-threshold information on depressive disorders and mental health in children and adolescents. The training was conducted synchronously online by trained psychologists. The training was delivered in small groups (mostly between 10–15 participants) to allow for interaction and discussion. The main content included (1) general information on depression (age specific symptoms, possible signs of depression in youths such as nonsuicidal self-injury, suicidality, the distinction between puberty and depression, symptoms of depression that could be recognisable in a school context, causes), (2) practical tips for supporting pupils with depression (information on how behavioural or emotional changes in pupils can be addressed in a school context, information on how educational professionals safely can manage situations with suicidal pupils, information about compensatory measures in the school context to support pupils in meeting academic requirements without being disadvantaged and communication tips) and (3) preventing depression and supporting pupils’ mental health in the school context (responding to school stressors such as pressure to perform, negative classroom climate and bullying, promoting a positive mindset, supporting mental health through sport and movement), including experience reports from affected pupils that were presented as videos or audio recordings. The focus of the training was on school-based strategies for supporting children and adolescents with depression as well as suicidal behaviour, and options for promoting mental health in the school context. In designing the training, several pedagogical aspects were taken into account to ensure high engagement. During the training, interaction with the participants was deliberately encouraged, for example by discussing previous experiences of the participants with pupils with mental health problems and methods for mental health promotion in the school context. Also, practical examples were discussed and debated. Different formats were used, including power point expert inputs, discussion rounds and short videos and audios including reports from youths with lived experience to maintain interest and facilitate learning. We applied some principles of andragogy [36] to ensure effective adult learning by designing sessions that were relevant, problem-oriented, and built on participants’ prior experiences. The content of the training was based on the websites “ich-bin-alles” (‘https://www.ich-bin-alles.de/’; English translation "I am everything”) and “ich-bin-alles @Schule” (‘https://schule.ich-bin-alles.de/’; English translation "I am everything @school”) developed by the Department of Child and Adolescent Psychiatry, Psychosomatics and Psychotherapy, LMU University Hospital, LMU Munich together with the Beisheim Foundation and media partners. The “ich bin alles” website is an evidence-based platform on major depression and mental health in children and adolescents for youths and parents, that was evaluated in several studies in these target groups [37–39]. The “ich bin alles @Schule” website is an extension for educational professionals providing information on depression in youths. The evidence base is included on the corresponding websites. The online training was intended to reach educational professionals who preferred an interactive way of learning and to provide the opportunity for active engagement and exchange. To address this need, the training was developed in addition to the website.

A pilot exploratory survey of educational professionals (*N* = 33), who had already taken part in the training course before the beginning of the study, showed positive results in terms of relevance, information content, transfer and the didactic concept, as well as a high level of perceived need among the target group. Feedback of the educational professionals on the training was incorporated in the training concept before the start of the evaluation study.

### Primary outcome

#### Assessment of knowledge

In order to assess knowledge changes regarding depression and mental health, a self-designed questionnaire which was based on the content taught in the training course, was used. The questionnaire was presented to the participants at all three assessment points (pre-, post- and four-week follow-up). The knowledge questionnaire consisted of 18 items in total, of which 15 were multiple-choice items (each with 4 answer options, with between 1 and 4 being correct) and 3 were single-choice items (each with 4 answer options, of which only 1 was correct). The knowledge questionnaire included six questions on the symptoms of depression in children and adolescents, six questions on supporting pupils with depression and school-related stressors, and six questions on the prevalence and causes of depression (see Table 1 for an overview and item examples). The answers were counted as correct if the entire question was answered correctly (correct = 1, incorrect = 0, total sum score: 0–18).

**Table 1:** Overview of the knowledge questionnaire with example questions

Domain	Number of items	Example item	Answer options
Symptoms	6	What can be signs of depression?	*a) lack of self-confidence b) frequent crying* c) repetitive, stereotypical behaviour (e.g., flapping arms) *d) withdrawal from peers*
Supporting pupils with depression and stressors at school	6	In order to reduce the pressure on pupils to perform, teachers can…	a)…completely avoid constructive criticism *b)…pay attention to stress compensation and address self-reinforcement and resources c)…communicate performance expectations transparently and clearly* d)…do nothing, as the pressure comes mainly from the parents and the pupils themselves
Frequency and cause	6	What applies to depression in adolescence?	a) A person can only be said to have a “real depression” if they have had more than one depressive episode b) Depression usually only occurs once *c) 50–70% of adolescents suffer from a recurrent depressive episode within 5 years d) During the coronavirus pandemic*,* the number of adolescents exhibiting depressive symptoms doubled*

Correct answers are indicated in italics

#### Assessment of stigma

Stigma towards people with depression was measured using the German version of the Personal Stigma Subscale of the Depression Stigma Scale (DSS; [40, 41]). The internal consistency of the questionnaire in our study was high (Cronbach’s alpha = 0.89). Participants were presented with nine common prejudices about people suffering from depression (e.g., “Depression is not a real medical illness.”) and asked to respond on a five-point scale: “strongly agree (4), agree (3), neither agree nor disagree (2), disagree (1), strongly disagree (0)”. A higher total score (0–36) indicates stronger prejudice against people with depression. The questionnaire was administered to participants at all three assessment points (pre-, post- and four-week follow-up).

### Secondary outcome

#### Assessment of confidence/helping behaviour

To assess changes in participants’ confidence in supporting pupils suffering from depression and their behavioural intention to help, participants were asked how confident they felt in recognising, approaching, referring, and supporting pupils with depression. Responses were given on a five-point scale: not at all (0), a little bit (1), moderately (2), quite a bit (3), and very (4). Higher overall values (0–16) indicate greater confidence in own actions. The questions were based on assessments in similar studies on mental health teacher training courses [42–44]. The questionnaire was administered at all three assessment points. The internal consistency of this questionnaire in our sample was high (Cronbach’s alpha = 0.86).

To measure helping behaviour, participants were asked at the pre- and follow-up assessment, whether they had spoken to a pupil with mental health problems about psychological issues in the last four weeks based on Jorm [43]. If so, they were asked about the subject of their conversation (e.g., spending time to help the pupil to calm down, advising them to seek professional help).

The post-assessment also assessed the extent to which participants planned to apply the content of the training course in their daily school life (answer options: not at all true, somewhat true, mostly true, fully true). Additionally, the follow-up assessment examined to what extent participants had already implemented the content and, if so, how, recorded using an open-text field.

#### Assessment of the quality of the training

The quality of the training (e.g., didactics, information content) was evaluated using self-designed questions (for details see Supplement table A.1). The questions related to the participants’ interest in the training, previous knowledge, the perceived information content and practical relevance. Other questions included information on the structure of the training course, its length, opportunities for participants to ask questions and the perceived way in which the lecturer conducted the course. There was also the opportunity to provide open feedback. The overall rating of the participants for the training was 1.9 (on a German grading scale from 1 = very good to 6 = insufficient).

### Procedure

Study recruitment and assessment took place from April 2024 until September 2024. Study information was sent via e-mail. Participants were provided with a written explanation of the study’s aims and objectives. Informed consent was obtained from the participants via selecting “I agree to the data processing”. To register for the study, respondents entered their email address under a link they received (via the online survey tool LimeSurvey). After registering for the study, participants received a link to the pre-assessment via e-mail, one day before taking part in the two-hour online training session. During the pre-assessment time point, participants created an individual code to ensure anonymous linkage of the three assessment time points. Each assessment took about 20 min. The total duration of the study, including the training, was about 3 h. After completing the follow-up assessment, participants had the opportunity to enter their e-mail address on a separate page, due to anonymity, to receive the expense allowance (75€ bank transfer/Amazon voucher). The assessment instruments and the respective assessment dates are shown in Table 2.

**Table 2:** Study plan

Function	Measure	Time points	Assessment instruments
Inclusion and exclusion criteria	Sociodemographics	Pre	Self-developed questionnaire
Primary outcome measure	Knowledge of depression symptoms, its prevalence, causes, and how to support pupils with depression and stressors in the school environment	Pre, post, follow-up	Self-developed questionnaire based on training content
	Stigma regarding depression	Pre, post, follow-up	Personal Stigma Subscale of the Depression Stigma Scale (DSS)
Secondary outcome measure	Behavioural confidence	Pre, post, follow-up	Questions from Sebbens, 2016
	Helping behaviour	Pre, follow-up	Question from Jorm, 2010
Additional measures	Evaluation of the training	Post	Self-developed questionnaire

pre = 0–1 days before training, post = 0–1 day after training, follow-up = 4 weeks after training; *N*pre = 97; *n*post = 95 for knowledge; *n*post = 94; *n*follow-up = 79

### Power analysis

The primary outcome variables were knowledge and stigma change, measured at pre-, post- and follow-up intervention time points. The a priori calculation of the sample size was optimised with regard to the changes from pre- to follow-up. Previous studies on teacher training on mental health reported medium to large effects in pre-follow-up designs (knowledge increase Cohen’s d = 1.10–1.28; stigma change Cohen’s d = 0.68 − 1.00; [45, 46]). We adopted a conservative approach, anticipating small to medium effects since our intervention was shorter in comparison to the interventions described in the previous papers. In order to achieve this effect with an alpha error = 0.05 and a power of 0.80, *N* = 42 people must be included. With an estimated dropout rate of 20%, this results in a sample of *N* = 50 participants. Due to high number of registrations and strong interest in the training, a larger sample was included. The sample size was calculated using G*Power 3.1.9.2.

### Data analysis

Statistical data analysis was carried out using IBM SPSS Statistics 29. The significance level was set to *p* = .05, two-tailed. In order to analyse knowledge acquisition, stigma change and confidence in one’s own abilities in supporting pupils with depression (assessed pre, post, and follow-up), paired *t*-tests were conducted. According to Cohen’s *d* [47], effect sizes can be classified as follows: 0.20 = small effect; 0.50 = medium effect; 0.80 = large effect.

To control for effects of possible website consumption after the training, a question was added during the study asking whether participants have visited the websites “ich-bin-alles” or “ich-bin-alles @Schule” between the training and follow-up assessment. This question was added mid-study and therefore was presented to 44 out of 79 follow-up respondents. Exploratory sensitivity analyses were calculated (main analyses were repeated) for the primary outcome measure knowledge acquisition for those who reported having visited the website (*n* = 18) and those who reported not having visited the website (*n* = 26), see Supplement Table A.3.

## Results

### Knowledge acquisition

**Fig. 1:**
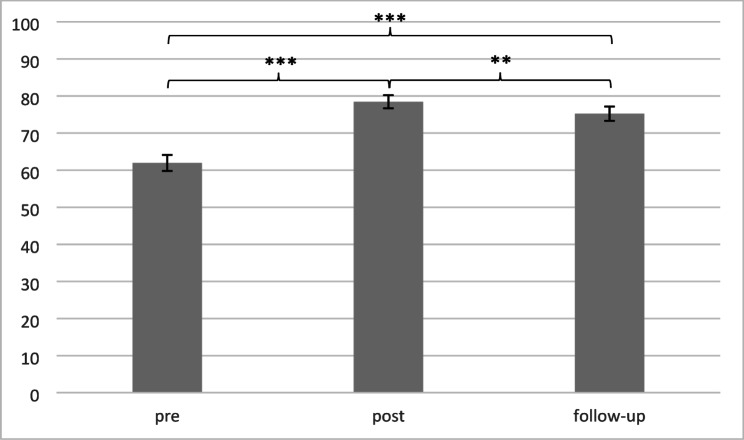
Mean knowledge change over time in % (SE); **p < .01; ***p < .001

Paired *t*-tests revealed a significant increase in knowledge from pre to post *t*(94) = 12.29, *p < .*001, Cohen’s *d* = 2.33. Although we found a decrease from post to follow-up *t*(76)=-2.80, *p = .*007, Cohen’s *d* = 2.25, the increase in knowledge was still significant from pre to follow-up *t*(78) = 6.80, *p < .*001, Cohen’s *d* = 3.00 (see Fig. 1, means can be found in Table A.2).

### Stigma change

**Fig. 2:**
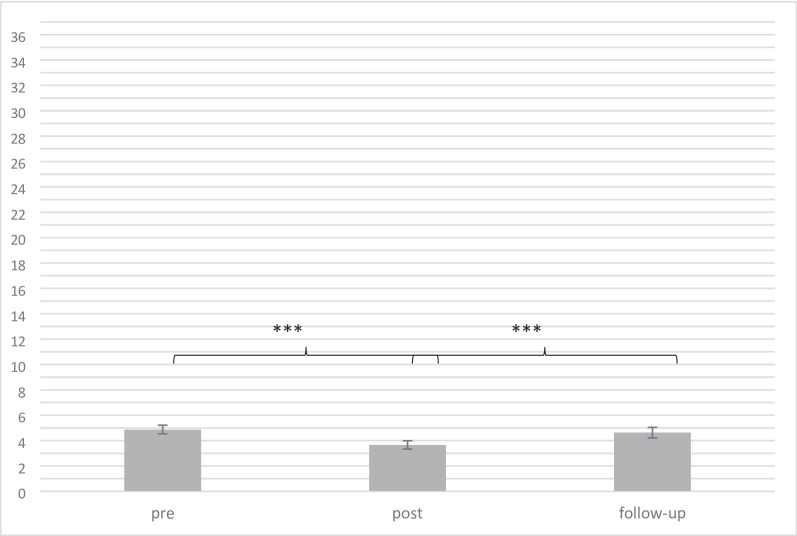
Changes in stigma over time (*M*, *SE*); ***p < .001

Paired *t*-test from pre to post showed a significant reduction in the stigma score *t*(93)=-5.33, *p <* .001, Cohen’s *d* = 2.34. Mean differences from post- to follow-up assessment showed an elevation in the stigma score *t*(75) = 3.33, *p <* .001, Cohen’s *d* = 2.62. No significant effect was revealed from pre- to follow-up assessment *t*(78)=-0.91, *p* = .367, Cohen’s *d* = 2.85 (see Fig. 2, for more details see Table A.2).

### Changes in confidence

**Fig. 3:**
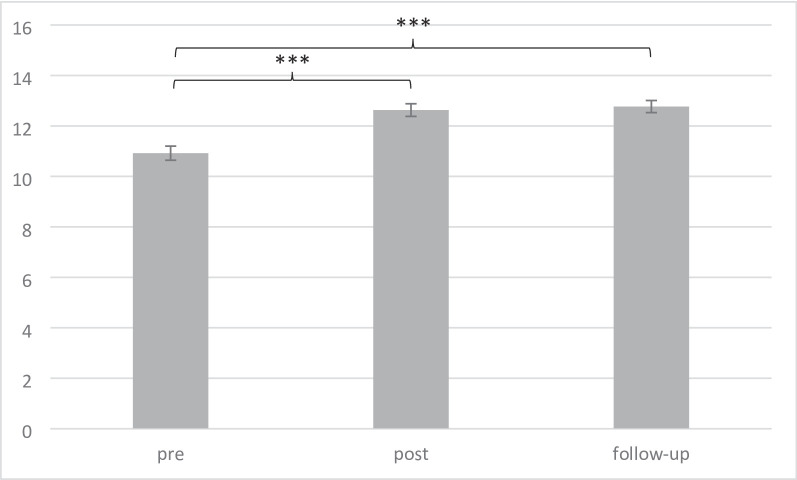
Changes in confidence over time (*M*, *SE*); ***p < .001

Paired *t*-tests from pre to post, *t*(93) = 7.00, *p < .*001, Cohen’s *d* = 2.45, and from pre to follow-up, *t*(78) = 7.85, *p <* .001, Cohen’s *d* = 2.12, showed a significant elevation in confidence. No decrease was observed from post- to follow-up assessment, *t*(75)=.-28, *p* = .78, Cohen’s *d* = 1.64 (see Fig. 3, for more details see Table A.2).

### Change in helping behaviour

Before the training 58.8%, and at follow-up 69.6% of the participants report that they spoke to a pupil in the past four weeks regarding their mental health problems. A McNemar test was conducted to examine changes in reported helping behaviour between pre- and follow-up assessment *χ²*(1, *n* = 79) = 8.78, *p* = .108, suggesting no significant change (for more information see Table 3).

**Table 3:** Reported and intended helping behaviour

Helping behaviour	Pre (*N* = 97)	Follow-up (*N* = 79)
Did you talk with a pupil about their mental health problems in the last four weeks?	Yes: *n* = 57 (58.8%) No: no further questions were asked	Yes: *n* = 55 (69.6%) No: no further questions were asked
If yes: Did you spend time listening to their problems?	Yes: *n* = 52 (91.23%)	Yes: *n* = 52 (94.5%)
If yes: Did you help them to calm down?	Yes: *n* = 31 (54.39%)	Yes: *n* = 37 (67.3%)
If yes: Did you talk to them about suicidal thoughts?	Yes: *n* = 16 (28.07%)	Yes: *n* = 15 (27.3%)
If yes: Did you advise them to seek professional help?	Yes: *n* = 33 (57.89%)	Yes: *n* = 40 (72.7%)
If yes: Other (open-text field)?	Yes: *n* = 11 (19.30%)	Yes: *n* = 5 (9.1%)

Intended behaviour	Post (*N* = 94)	Follow-up (*N* = 79)
“I intend to apply the content of the training course in everyday school life in the future”	Yes: *n* = 40 (42.55%)	Yes: *n* = 44 (55.70%)
“I have already used the contents of the training course in everyday school life”	n.a.	Yes: *n* = 19 (24.05%)

### Sensitivity analyses for website visit

Exploratory sensitivity analyses were calculated in a subsample for those who reported having visited the website (*n* = 18) and those who reported not having visited the website (*n* = 26). The results in the two subsamples resembled the results in the whole sample with one exception. Similar to the results in the whole sample, participants who did not visit the website after the training showed a significant reduction in knowledge from post to follow-up, *t* (25)=-3.43, *p* = .002, Cohen’s *d* = 1.66. Unlike in the main sample, those who visited the website showed no significant reduction in knowledge from post to follow-up, *t*(16)=-1.61, *p* = .127, Cohen’s *d* = 2.26. More details can be found in the Supplement (Table A.3).

## Discussion

The study aimed to examine whether a two-hour online training course for educational professionals on depression and mental health in childhood and adolescence leads to an increase in knowledge, reduction of stigma, increase in confidence in supporting pupils with depression and increase in helping behaviour. With regard to knowledge acquisition, we observed an increase in knowledge after the training course which was still significant at the follow-up four weeks later. With regard to change in stigma towards depression, there was a reduction in stigma from pre- to post-training, which returned to its baseline from post to follow-up. Confidence in supporting pupils with depression increased from pre- to post-training and remained stable over time. No significant change in reported helping behaviour was found. The structure, format, content and didactics of the training were rated positively by the participants.

In line with previous studies [23, 24] and consistent with the hypotheses, an increase in knowledge was obtained and remained significant in the follow-up assessment four weeks later. Previous training programmes were predominantly designed for secondary school settings, limited to teaching staff, and often involved resource-intensive, in-person delivery formats [27]. In our study, we implemented a more inclusive and scalable approach, with the online training format enabling knowledge gains across various school settings and among different educational professionals. A slight decrease in knowledge was observed from post to follow-up. The short term knowledge loss aligns with findings from prior research [48] and is consistent with the classic Ebbinghaus forgetting curve, which demonstrates that information is quickly forgotten over time, with the most significant loss occurring shortly after learning [49, 50]. The sensitivity analysis revealed that participants who revisited the material —by accessing the thematically relevant website(s) “ich-bin-alles (@Schule)”— within four weeks after the training demonstrated no significant retention loss unlike those who did not revisit the material. This suggests that brief knowledge refreshers are beneficial in mitigating the forgetting phenomenon [51]. Future training programmes should consider integrating structured repetition exercises to enhance long-term knowledge retention.

The training achieved a short-term reduction in stigma after the training course, which however returned to the baseline level after four weeks. When discussing the data concerning change in stigma, several aspects should be taken into account. The sample consisted of educational professionals who signed up voluntarily for the training and who had already taken part in a MHFA course, and therefore had prior experience on mental disorders. Around one quarter of the participants were school social workers and school social pedagogues who support children and adolescents with mental health problems on a daily basis. Although our pre-post-follow-up-design aimed at evaluating only our training course and not MHFA, it should be acknowledged that our total sample showed a relatively low mean stigma towards people with depression at pre-test compared to the general population [52], which might be related to the prior MHFA course. This floor effect could have limited the ability to capture the full potential of the training with regard to stigma change. Since anticipated stigmatization is a major factor that may prevent individuals from seeking help [15], the data suggest that the participation of educational professionals in the training can help reduce stigma in the short term, but ongoing reinforcement is necessary.

On the one hand, no significant changes in reported helping behaviour were found. However, it should be noted that the follow-up period spans only four weeks, which is a relatively short timeframe with possibly only limited opportunities for the active application of the acquired knowledge (see also [43]). On the other hand, confidence in participants’ own abilities in supporting pupils with depression increased through training and remained stable over time, which is in line with previous studies [19, 31, 44]. Research has indicated that mental health literacy and self-confidence in one’s abilities may serve as predictors of helping behaviour [22]. Irrespective of helping behaviour, a quarter of the participants reported at follow-up that they had already applied some of the acquired content. In the future, more research should be focused on tracking helping behaviour before and after a training, for example by using ecological momentary assessment (EMA).

The acceptance of the two-hour online training format was high. The results suggest that trainings with practical intervention instructions such as ‘How should I approach a pupil who I suspect is suffering from mental health problems?’ and preventive school exercises, such as ‘How can I promote a positive attitude at school?’ result in strong approval from the target group. The cost and time efficient format enabled educational professionals from a wide range of school types, grade levels, and geographical locations to participate. The promising results may be transferable to other highly relevant school-related disorders like exam anxiety or learning disorders. Knowledge about a wide range of disorders could result in increased confidence of educational professionals in their own ability to support pupils with mental health problems, which in turn may lead to greater helping behaviour.

The school setting contains a multitude of potential stressors and simultaneously is the place where children and adolescents spend most of their everyday life. Support options are usually initiated too late [53], that is why various prevention programmes are developed. However, recently the risks of adverse effects of pupil-based approaches on mental health like universal programmes have been discussed. Studies show an increase in internalising symptoms in subgroups of adolescents, that could be explained by reinforcement of distress through psychological labelling, and amplifying negative experiences via peer influence and social learning in group settings (for further discussion see [54]). These adverse effects could be avoided through focusing on teacher trainings while still achieving positive effects on adolescents’ mental health. By destigmatizing distress and mental health problems among teachers, and by guiding teachers to promote emotional and social skills integrated into everyday school life, a more positive and supportive school environment could be created.

### Limitations and conclusions

Certain limitations of the study should be taken into account. We employed a single armed pre-post-follow-up design with no control group, which does not exclude the possibility that the observed changes are not solely attributable to the intervention itself, but other factors such as natural fluctuations, external influences, or participants’ exposure to related information might have played a role as well. Selection of participants may be influenced by factors, such as high interest and knowledge in this topic and lower stigma levels. This may limit the generalizability of the findings to the broader population of education professionals. Future research should aim to assess the impact of similar training courses in randomized controlled trials to assess the efficacy of these trainings. In the present study, engagement during the training was not formally measured beyond attendance, participants were encouraged to turn on their camera during the training and self-reported interest in the training course was assessed. For future studies, engagement could be systematically captured using measures such as active participation tracking (e.g., responses to exercises or interactive talks), or observational coding by trainers. Implementing such measures would allow a more precise assessment of participants’ involvement throughout the training. In addition, the control question whether participants had visited the “ich-bin-alles” or “ich-bin-alles @Schule” websites, which cover a large portion of the training content, was added later in the course of the study, therefore sensitivity analyses could only be performed for a subsample. A further limitation is the short follow-up period with self-report assessments, which restricts the ability to detect behavioural change like helping behaviour and talking about pupils’ suicidal thoughts. Future studies should include longer follow-ups with the collection of behavioural and more objective data to be able to draw more comprehensive conclusions about the effects of the training. Furthermore, the study was limited to German-speaking participants within the context of the German school system. Consequently, in order to transfer the findings to other linguistic and educational contexts, further adaptions would be required.

Despite these limitations, the study highlights several key strengths that underscore the success of the training course. The two-hour online format was time- and cost-efficient and ensured accessibility for a diverse group of participants while achieving promising outcomes. Participants expressed high acceptance of the training’s structure and format. The follow-up design provided information regarding the sustainability of the effects. Importantly, the training showed measurable effects not only for teachers but also for social workers and school social pedagogues, illustrating its relevance and applicability across various professional fields. Our findings suggest that periodic knowledge boosters may be beneficial to sustain these positive outcomes over time and counteract potential retention loss. Moreover, such courses could pave the way for efficiently and successfully training educational professionals to recognise and respond to pupils’ mental health challenges, thereby possibly helping to close the treatment gap for conditions like youth depression.

## Supplementary Information

Supplementary material 1.

## Data Availability

The datasets used and/or analysed during the current study are available from the corresponding author on reasonable request.

## Abbreviations

DSS: Depression Stigma Scale
EMA: Ecological momentary assessment
M: Mean
MHFA: Mental Health First Aid
N/ n: Number of participants
p: Probability value
SD: Standard deviation
SE: Standard error

## Declarations

### Other

During the preparation of this work the authors used ChatGPT in order to spell- and grammar-check parts of the manuscript. After using this tool/service, the authors reviewed and edited the content as needed and take full responsibility for the content of the publication.
