# Supplementary material for: Evaluation of an online training course for educational professionals on depression and mental health in childhood and adolescence
Source: Child Adolesc Psychiatry Ment Health. 2025 Dec 15;20:8. doi: 10.1186/s13034-025-01007-y (PMC12822316; doi:10.1186/s13034-025-01007-y)
Supplement: Supplementary file 1 — Supplementary material 1. [file 13034_2025_1007_MOESM1_ESM.pdf]

Table A.1

Results of the assessment of the quality of the training (in %)

Items	Response option 1	Response option 2	Response option 3	Response option 4	Response option 5	Response option 6
How high was your interest in the “training on depression and mental health in schools” at the beginning of the event?	Very low (0)	Low (1.1)	Medium (7.4)	High (42.6)	*Very high (48.9)*	
How much prior knowledge did you have in this subject area?	Very low (0)	Low (2.1)	*Medium (64.9)*	High (27.7)	Very high (5.3)	
The information content of the “training on depression and mental health in schools” was:	Very low (0)	Low (1.1)	Medium (19.1)	*High (60.6)*	Very high (19.1)	
The length of the “training on depression and mental health in schools” was:	Far too short (2.1)	Slightly too short (24.5)	*Just right (68.1)*	Slightly too long (5.3)	Far too long (0)	
The scope of the content of the “training on depression and mental health in schools” was:	Far too little (3.2)	Slightly too little (35.1)	*Just right (60.6)*	Slightly too big (1.1)	Far too big (0)	
The number of slides was:	Far too small (0)	Slightly too small (10.6)	*Just right (88.3)*	Slightly too large (1.1)	Far too large (0)	
The “training on depression and mental health in schools” has a high practical relevance for me:	Strongly disagree (1.1)	Somewhat agree (14.9)	*Mostly agree (53.2)*	Strongly agree (30.9)		
In the future, I plan to apply the content of the “training on depression and mental health in schools” in everyday school life:	Strongly disagree (0)	Somewhat agree (14.9)	*Mostly agree (42.6)*	*Strongly agree (42.6)*		
The slides were well-designed and visually appealing	Strongly disagree (1.1)	Somewhat agree (13.8)	*Mostly agree (50.0)*	Strongly agree (35.1)		

The content of the training was well structured	Strongly disagree (0)	Somewhat agree (3.2)	Mostly agree (40.4)	*Strongly agree (56.4)*		
I had the opportunity to ask questions	Not at all true (0)	Somewhat true (1.1)	Mostly true (10.6)	*Fully true (88.3)*		
The lecturer expressed herself clearly and comprehensibly	Not at all true (0)	Somewhat true (2.1)	Mostly true (12.8)	*Fully true (85.1)*		
The lecturer made the course engaging and diverse	Strongly disagree (0)	Somewhat agree (10.6)	Mostly agree (34.0)	*Strongly agree (55.3)*		
How would you rate the overall impression of the “training on depression and mental health in schools”?	Insufficient (0)	Poor (1.1)	Sufficient (3.2)	Satisfactory (9.6)	*Good (59.6)*	Very good (26.6)

Note: n=94; the highest percentage is written in italics.

Table A.2

Questionnaire data

**Variable/*M*(*SD*)<br>*M*(*SD*)%**	**Pre**	**Post**	**Follow-up**
Knowledge	11.15 (2.40)<br>61.94 (13.33)%	14.12 (2.42)<br>78.44 (13.44)%	13.54 (2.29)<br>75.22 (12.72)%
Stigma	4.87 (3.35)	3.66 (3.23)	4.63 (3.77)
Confidence	10.92 (2.78)	12.63 (2.41)	12.77 (2.10)

*Note: N*pre=97; *n*post=95 for knowledge; *n*post=94; *n*follow-up=79.

Table A.3

Sensitivity analyses paired t-test

This table presents exploratory sensitivity analyses for the two primary outcome measures (knowledge and stigma) and the secondary outcome (confidence) separately for those who reported having visited the website (*n*=18) and those who reported not having visited the website (*n*=26).

**Model**	***t*-value (pre-post)**	***p*-value (pre-post)**	**Effect size *d* (pre-post)**	***t*-value (post-follow-up)**	***p*-value (post-follow-up)**	**Effect size *d* (post-follow-up)**	***t*-value (pre-follow-up)**	***p*-value (pre-follow-up)**	**Effect size *d* (pre-follow-up)**
**Knowledge** (based on full sample as reported in the manuscript)	12.29	<.001* (post > pre)	2.33	-2.80	.007* (post > follow-up)	2.25	6.80	<.001* (follow-up > pre)	3.00
Model 1: Visited website[1]	3.95	.001* (post > pre)	2.58	-1.61	*.127*	2.26	2.74	.014* (follow-up > pre)	2.76
Model 2: Did not visit website[2]	6.46	<.001* (post > pre)	2.55	-3.43	.002* (post > follow-up)	1.66	3.81	<.001* (follow-up > pre)	2.83

*Note: N*pre=97; *n*post=95 for knowledge; *n*follow-up=79; *significance level was set to <.05; [1] *n* pre-post and post-follow-up=17, *n* pre-follow-up=18; [2]*n*=26; The difference revealed in the sensitivity analyses compared to the original model is written in italics.
